# Circulating miR-148b and miR-133a as biomarkers for breast cancer detection

**DOI:** 10.18632/oncotarget.2014

**Published:** 2014-05-26

**Authors:** Jie Shen, Qiang Hu, Michael Schrauder, Li Yan, Dan Wang, Leonardo Medico, Yuqing Guo, Song Yao, Qianqian Zhu, Biao Liu, Maochun Qin, Matthias W. Beckmann, Peter A. Fasching, Reiner Strick, Candace S. Johnson, Christine B. Ambrosone, Hua Zhao, Song Liu

**Affiliations:** ^1^ Department of Epidemiology, the University of Texas MD Anderson Cancer Center, Houston, TX, USA; ^2^ Department of Biostatistics & Bioinformatics, Roswell Park Cancer Institute, Buffalo, NY, USA; ^3^ Department of Cancer Prevention and Control, Roswell Park Cancer Institute, Buffalo, NY, USA; ^4^ Department of Pharmacology and Therapeutics, Roswell Park Cancer Institute, Buffalo, NY, USA; ^5^ Institute of Basic Medical Sciences, Chinese Academy of Medical Sciences, School of Basic Medicine, Peking Union Medical College, Beijing, P.R.China; ^6^ Department of Gynecology and Obstetrics, University Hospital Erlangen, Friedrich-Alexander University Erlangen-Nuremberg, Erlangen, Germany

**Keywords:** circulating microRNAs, breast cancer, biomarkers, detection

## Abstract

Circulating microRNAs have drawn a great deal of attention as promising novel biomarkers for breast cancer. However, to date, the results are mixed. Here, we performed a three-stage microRNA analysis using plasma samples from breast cancer patients and healthy controls, with efforts taken to address several pitfalls in detection techniques and study design observed in previous studies. In the discovery phase with 122 Caucasian study subjects, we identified 43 microRNAs differentially expressed between breast cancer cases and healthy controls. When those microRNAs were compared with published data from other studies, we identified three microRNAs, including miR-148b, miR-133a and miR-409-3p, whose plasma levels were significantly higher in breast cancer cases than healthy controls and were also significant in previous independent studies. In the validation phase with 50 breast cancer cases and 50 healthy controls, we validated the associations with breast cancer detection for miR-148b and miR-133a (P = 1.5×10^−6^ and 1.3×10^−10^, respectively). In the *in-vitro* study phase, we found that both miR-148b and miR-133a were secreted from breast cancer cell lines, showing their secretory potential and possible tumor origin. Thus, our data suggest that both miR-148b and miR-133a have potential use as biomarkers for breast cancer detection.

## INTRODUCTION

microRNAs are a class of small noncoding RNAs that play a central role in the regulation of mRNA expression [1-5]. The discovery that microRNA expression is frequently dys-regulated in a cancer-specific manner provides an opportunity to develop these RNAs as biomarkers for cancer detection [6-12]. Most previous studies on microRNA expression have been performed on tissue specimens. However, because tumor-derived microRNAs can be present in blood and appear to be stable to certain degree and protected from endogenous ribonuclease activity in circulation, some studies have shown diagnostic and prognostic potential for circulating microRNAs [13-25].

The potential of circulating microRNAs as biomarkers for cancer early detection is particularly relevant to breast cancer because it is the most common cancer in US women, regardless of race or ethnicity, despite improvement in cancer screening and treatment strategies. Mammography is the current gold standard, but can have false negative rates of up to 20% [26]. The diagnosis of breast cancer relies on the histological examination of tissue biopsies, or cytology of fine-needle aspirates, which are both invasive procedures. Known serum-based tumor markers, such as CA15.3 and BR27.29, cannot be used for breast cancer detection due to low sensitivity. There is thus a need for developing novel markers that are minimally invasive, for the improved detection of breast cancer.

Previously, our group, as well as others, have compared the profiles of circulating microRNAs between breast cancer patients and healthy controls, and attempted to identify circulating microRNA-based breast cancer detection biomarkers [16, 19, 20, 22, 23, 25, 27-52]. Although a number of circulating microRNAs have been identified, the results are widely inconsistent. To date, no consistent diagnostic signature for circulating microRNAs in breast cancer is available. Several issues, including patient heterogeneity, microRNA contamination from various blood components, microRNA quantification platforms, data normalization, and biological significance are likely contributors to the inconsistency [9, 32, 53]. Thus, a study to appropriately address those issues is highly desirable.

In light of these observations, in the first phase, we performed quantitative PCR-based plasma microRNA profiling analysis among invasive breast cancer patients, patients with ductal carcinoma in situ (DCIS), and healthy women. In the second phase, we compared the significant microRNAs identified from our study with other published studies and then validated those with confirmed associations in independent samples. In the third phase, we determined whether cultured breast cancer cell lines secreted those validated circulating microRNAs into the culture medium, attempting to understand the possible origins of those identified microRNAs.

## RESULTS

In the discovery cohort, a total of 122 women, including 52 with invasive breast cancer, 35 with DCIS, and 35 healthy controls, were included in the analysis. All of the study subjects were Caucasians. The mean age of invasive breast cancer cases, DCIS cases, and controls were 52, 55, and 55 years, respectively. There was no statistically significant difference in age. All invasive breast cases had histologically confirmed early stage (I and II) invasive ductal carcinoma. Blood samples were drawn prior to surgery. The tumor size ranged between 0.2 to 2.5 cm. ER, PR and HER2/neu status data were available for 50 patients with invasive breast cancer: 39 ER+, 11 ER−; 41 PR+, 9 PR−; and 12 HER2/neu+, 38 HER2/neu−. Eight patients had triple negative breast cancer.

**Figure 1:**
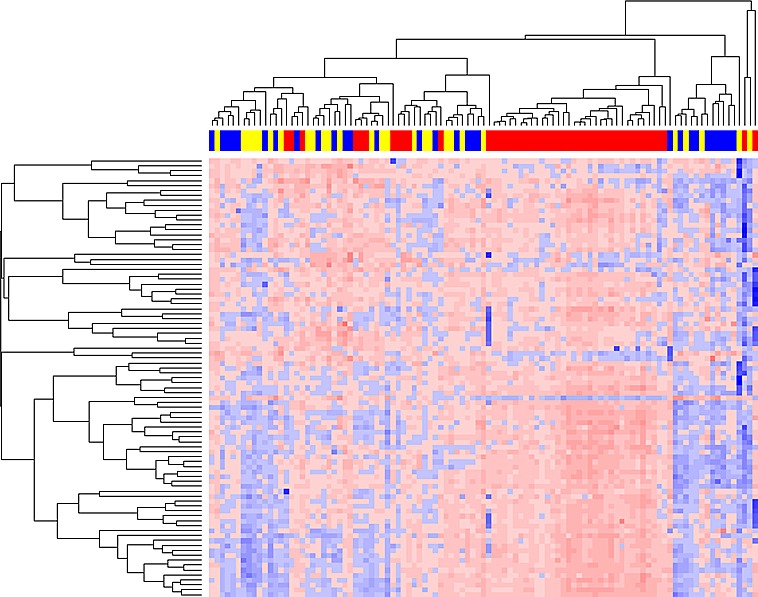
Unsupervised hierarchical clustering analysis of serum microRNA expression profiles In the clustering heat map, red indicates up-regulated while blue indicates down regulated. In the sample clustering dendrogram, red indicates invasive samples, blue indicates DCIS samples, while yellow indicates control samples.

Unsupervised hierarchical clustering analysis based on the top 75% of the most variable microRNAs across 106 samples passing quality control indicates that the microRNA expression profiles of invasive samples were intrinsically different from those of DCIS and control groups, while the microRNA expression profiles of DCIS and control groups were not different from each other. As shown in Figure 1, three major clusters exist (from right to left): the first cluster with predominately DCIS and control samples, the second cluster with all invasive samples, and the third cluster with a mixture of DCIS, control and invasive samples. A similar clustering pattern was observed when all microRNAs in the PCR panel were used (data not shown).

**Figure 2:**
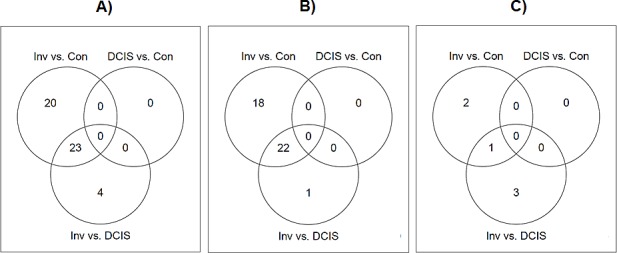
Venn diagrams showing the overlap of differentially expressed microRNAs identified from each of the three comparisons: invasive group versus control group, invasive group versus DCIS group, and DCIS group versus control group, respectively A) The overlap of all differentially expressed microRNAs; B) The overlap of up-regulated differentially expressed microRNAs; C) The overlap of down-regulated differentially expressed microRNAs.

Pair-wise comparisons between invasive, DCIS and control groups were performed in order to identify differentially expressed microRNAs with at least 2-fold expression change at the significance level of FDR <0.01. In consistent with the clustering analysis, no differentially expressed microRNAs were found between DCIS and control groups, and the expression levels of a number of microRNAs were found to be significantly elevated in the invasive samples compared with DCIS or controls. For the comparison between invasive and control groups, we identified 43 differentially expressed microRNAs, with 40 up-regulated in invasive samples and 3 down-regulated. For the comparison between invasive and DCIS groups, we identified 27 differentially expressed microRNAs, with 24 up-regulated in invasive samples and 3 down-regulated. The detailed list of differentially expressed microRNAs is summarized in Table 1. As shown in Figure 2, the majority (23 out of 27) of differentially expressed microRNAs identified from invasive versus DCIS comparison are included in the list of differentially expressed microRNAs identified from invasive versus control comparison.

**Table 1:** Differentially expressed microRNAs identified from Invasive versus Control/DCIS comparisons

Invasive versus Control				Invasive versus DCIS			
ID	FC	FDR	AUC	ID	FC	FDR	AUC
hsa-miR-136	19.63386	1.27x10^−08^	0.867218	hsa-miR-136	18.59352	7.70x10^−08^	0.88628
hsa-miR-199a-5p	14.33637	4.15x10^−12^	0.910559	hsa-miR-33a	9.180245	3.91x10^−05^	0.827586
hsa-miR-485-3p	9.954621	1.10x10^−08^	0.874704	hsa-miR-199a-5p	6.217108	5.35x10^−07^	0.892883
hsa-miR-33a	9.615268	2.47x10^−05^	0.789204	hsa-miR-326	5.678471	7.92x10^−06^	0.870139
hsa-miR-495	9.239414	2.72x10^−08^	0.853034	hsa-miR-130a	4.440197	1.48x10^−06^	0.882979
hsa-miR-543	8.382473	1.10x10^−08^	0.86643	hsa-miR-495	4.092802	2.79x10^−04^	0.799707
hsa-miR-326	6.789743	7.23x10^−07^	0.878251	hsa-miR-485-3p	3.689859	5.47x10^−04^	0.7854
hsa-let-7d	5.873482	1.25x10^−08^	0.888495	hsa-let-7d	3.640281	1.88x10^−05^	0.872707
hsa-miR-130a	4.630065	4.36x10^−07^	0.86643	hsa-miR-142-5p	3.54654	2.13x10^−05^	0.845194
hsa-miR-221	4.575009	9.21x10^−08^	0.841608	hsa-miR-543	3.374637	4.91x10^−04^	0.768525
hsa-miR-382	4.410125	3.24x10^−04^	0.720646	hsa-miR-1974	3.29879	2.03x10^−03^	0.812546
hsa-miR-199a-3p	4.346987	8.51x10^−05^	0.836091	hsa-miR-22	3.17841	7.24x10^−06^	0.827586
hsa-miR-22*	4.335605	7.43x10^−06^	0.839638	hsa-miR-22*	3.095461	5.04x10^−04^	0.781731
hsa-miR-766	4.213962	2.20x10^−08^	0.855004	hsa-miR-107	2.732845	7.24x10^−06^	0.855833
hsa-miR-339-5p	4.143662	1.10x10^−08^	0.866036	hsa-miR-221	2.720919	3.05x10^−04^	0.790536
hsa-miR-1974	3.999563	3.08x10^−04^	0.849094	hsa-miR-339-5p	2.67183	3.65x10^−05^	0.825385
hsa-miR-127-3p	3.912698	5.63x10^−04^	0.753743	hsa-miR-766	2.32762	5.74x10^−04^	0.787968
hsa-miR-409-3p	3.884377	7.75x10^−05^	0.778566	hsa-miR-423-3p	2.250449	3.91x10^−05^	0.810712
hsa-miR-151-5p	3.730705	1.11x10^−09^	0.877463	hsa-miR-151-5p	2.241882	5.21x10^−05^	0.80741
hsa-miR-133a	3.652671	2.82x10^−05^	0.795508	hsa-miR-331-3p	2.241426	8.03x10^−03^	0.717168
hsa-miR-99b	3.399696	4.13x10^−06^	0.808905	hsa-miR-32	2.237895	7.25x10^−03^	0.809978
hsa-miR-107	3.28739	1.11x10^−07^	0.865642	hsa-let-7f	2.195287	3.22x10^−03^	0.761555
hsa-miR-142-5p	3.286851	4.51x10^−05^	0.816785	hsa-miR-28-5p	2.025899	8.11x10^−04^	0.768158
hsa-let-7f	3.278603	9.59x10^−06^	0.810481	hsa-miR-342-3p	-2.00785	3.24x10^−04^	0.787601
hsa-miR-139-5p	3.274487	4.18x10^−06^	0.789992	hsa-miR-140-3p	-2.05119	8.04x10^−03^	0.862436
hsa-miR-28-5p	3.137736	1.47x10^−07^	0.845942	hsa-miR-720	-2.46154	2.33x10^−04^	0.780998
hsa-miR-22	2.922818	1.91x10^−05^	0.845548	hsa-miR-144*	-3.72644	7.05x10^−07^	0.936537
hsa-miR-423-3p	2.545573	2.70x10^−06^	0.816391				
hsa-miR-335	2.519429	5.94x10^−03^	0.732467				
hsa-miR-328	2.446737	7.08x10^−06^	0.803783				
hsa-miR-23b	2.367462	9.18x10^−03^	0.763593				
hsa-miR-30c	2.33765	4.90x10^−04^	0.767139				
hsa-miR-484	2.312884	9.74x10^−05^	0.841608				
hsa-miR-331-3p	2.267972	5.81x10^−03^	0.71119				
hsa-mir-148b	2.166974	1.96x10^−05^	0.810087				
hsa-miR-339-3p	2.131754	6.73x10^−05^	0.760441				
hsa-miR-152	2.073642	2.40x10^−03^	0.746257				
hsa-miR-126	2.052121	2.78x10^−05^	0.774232				
hsa-miR-374a	2.04563	4.35x10^−03^	0.751379				
hsa-miR-30b	2.015793	3.24x10^−04^	0.764381				
hsa-miR-194	-2.12943	2.27x10^−03^	0.806147				
hsa-miR-375	-2.77381	3.40x10^−03^	0.739559				
hsa-miR-144*	-3.74132	3.48x10^−07^	0.94011				

When comparing between the invasive breast cancer and the healthy control group, the most significantly differentially expressed microRNA was miR-199a-5p. The plasma levels of miR-199a-5p were more than 14-fold higher in patients with invasive breast cancer than in healthy controls. After adjusting for multiple comparisons, the P value is 4.1×10^−12^. We also performed receiver operating characteristic curve (ROC) analysis for each differentially expressed microRNA. The areas under the curve (AUC) for miR-199a-5p was 0.91, indicating clear separation between the invasive breast cancer group and the healthy control group from our discovery cohort (Table 1). When comparing between invasive breast cancer and DCIS groups, the most significantly differentially expressed microRNA was miR-136. The plasma levels of miR-136 were more than 18-fold higher in patients with invasive breast cancer than in healthy controls. After adjusting for multiple comparisons, the P value reached 7.7×10^−8^. The AUC for miR-136 was 0.89, indicating clear separation between invasive breast cancer and DCIS groups in our discovery cohort (Table 1).

**Figure 3:**
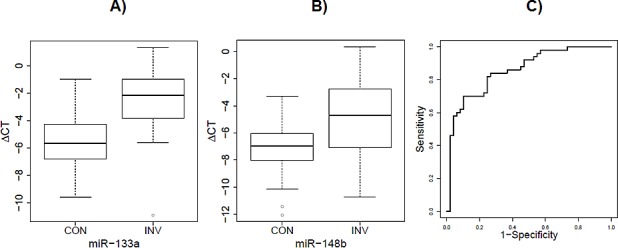
Elevated serum level of of miR-133a and mir-148b in invasive group versus control group of validation cohort A) The fold change of mir-133a was 8.3 (P = 1.3×10^−10^) in the validation cohort. B) The fold change of mir-148b was 5.1 (P = 1.5×10^−6^) in the validation cohort. C) The ROC curve derived from the combinations of these two miRNAs (AUC = 0.86) in the validation cohort.

We are mindful of the wide inconsistency among the previous circulating microRNA studies. Thus, we compared our results with others extracted from published studies [16, 19, 20, 22, 23, 25, 27-52], to determine if the significant microRNAs found in our studies were independently reproduced in any other studies. To ensure the quality, we restricted review to studies with at least one validation population. After an extensive literature search, none of our top significant microRNAs were reported previously in other studies. However, we found three microRNAs, namely miR-148b (fold change=2.17, adjusted P=1.96×10^−5^), miR-133a (fold change = 3.65, adjusted P=2.82×10^−5^) and miR-409-3p (fold change=3.88, adjusted P=7.75×10^−5^), which have also been reported to be up-regulated in circulation from breast cancer patients compared to healthy controls in other studies [38, 54]. Those three microRNAs were included in the validation stage analysis. Our validation cohort included a total of 50 patients with stage I and II breast cancer and 50 healthy controls. All study subjects were Caucasians. In this independent cohort, we found the plasma levels of mir-148b and miR-133a were also significantly higher in breast cancer patients than healthy controls (Figure 3). For mir-148b, the fold change was 5.1 (P = 1.5×10^−6^). For miR-133a, the fold change was 8.3 (P = 1.3×10^−10^). The AUC for the combination of these two microRNAs is 0.86. No significant association was found for miR-409-3p in the validation cohort.

**Figure 4:**
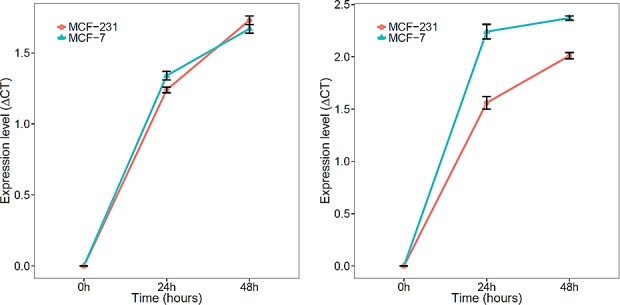
Expression of mir-148b and miR-133a in culture media of breast cancer cell lines (MCT-7 and MD-231) A) mir-148b levels in media of both MCF-7 and MD-231 increased with longer incubation intervals; B) miR-133a levels in media of both MCF-7 and MD-231 increased with longer incubation intervals.

Lastly, we determined whether miR-148b and miR-133a act as secretory microRNAs and are excreted into the culture media by MCF-7 and MDA-231 cell lines. We compared levels of miR-148 and miR133a in culture media among baseline (0 hr), 24 and 48 hrs. We observed both microRNA expression increased, suggesting that both microRNAs are possible secretory microRNAs (Figure 4).

## DISCUSSION

Published studies on circulating microRNAs as cancer detection biomarkers have identified a wide variety of microRNAs. Unfortunately, few of them have been validated in other studies. Recently, Leidner et al compared the circulating microRNAs results from several breast cancer studies [32] and were unable to demonstrate reproducibility by various measures across the different datasets. The widespread inconsistency may reflect technical difficulties, such as lack of standardization of biospecimens, quantification platforms, and internal controls. For example, the biospecimen type varies among studies. Some use plasma and serum, and some use whole blood. Serum and plasma are considered equivalent, although microRNA concentration appeared to be higher in serum [55]. The use of whole blood will lead to the isolation of microRNAs from many cell types including those within blood cells, but not just circulating microRNAs, warranting caution when comparing microRNA profiles derived from blood with those from serum or plasma [56]. As demonstrated by Pritchard et al, even serum and plasma samples may be contaminated with microRNAs from a variety of blood cells [56]. Such microRNAs may include mir-16, mir-150, mir-486-5p, let-7a, mir-574-3p, mir-223, mir-197, mir-451, and mir-92a. To be cautious, we did not include any of them in the analysis. The results may also vary by experimental approaches. So far, microarray profiling, next generation sequencing, quantitative RT-PCR profiling, or targeted assays of specific microRNAs have been employed in previous studies. Even with the same samples, the results from different platforms could be inconsistent, and RT-PCR, generally considered as the “gold standard” assay, is required to validate the discovery. In our current study, the RT-PCR approach was used in both discovery and validation cohort. There is also a problem about internal controls. To date, there is no consistent internal control microRNA available for normalizing circulating microRNA expression. The use of spike-in or a small RNA for data normalization in similar studies has sometimes been considered to be problematic due to their suspected instability. In the current study, because of using a larger profiling cohort, we had the capacity to select internal reference microRNAs empirically.

The inconsistency may also reflect the limitations stemming from study design. For example, most of the published studies do not have a validation population and the sample size for most of the studies is modest, thus patient heterogeneity becomes an ever bigger issue. To overcome those issues, the current study included both discovery and validation populations, both of which have adequate sample size. To further foster the consistency, we performed a literature review on circulating microRNAs and breast cancer, and compared our data with others [16, 19, 20, 22, 23, 25, 27-52]. Three microRNAs which were significantly different between breast cancer cases and controls in the discovery cohort of our study, namely mir-148b, miR-133a and miR-409-3p, were independently reproduced in other studies. miR-148b and miR-409-3p were reported previously by Cuk et al [38]. Using a two-step approach (discovery and validation), they found that 4 microRNAs (mir-148b, miR-376c, miR-409-3p and miR-801) were shown to be significantly up-regulated in the plasma of breast cancer patients in the validation cohort (N=127). Using three microRNAs (mir-148b, miR-409-3p and miR-801) in combination in ROC curve analysis, the discriminatory power reaches to 0.69 between breast cancer cases and healthy controls. miR-133a was reported in a recent study by Chan et al [54]. Using paired breast cancer tumor and normal tissues and serum samples, they found miR-133a, miR-133b, miR-1 and miR-92a were the most important diagnostic markers in the discovery cohort (N=32), which were then successfully validated (N=132). Results from our validation cohort confirmed the association with breast cancer detection for mir-148b and miR-133a.

One of the major problems about circulating microRNA research is the lack of an understanding of how those identified significant microRNAs enter the circulation. It has been hypothesized that most of the circulating microRNAs are scavengers of tumor cell apoptosis or necrosis. If that proves to be true, we expect to observe correlations between paired tumor tissues and serum/plasma. However, several recent studies have shown that the microRNA profiles between sera and the corresponding matched tumor are largely dissimilar, and circulating microRNAs do not reflect their abundance in the malignant cells [38, 54]. Those observations raise the possibility of the existence of alternative mechanism, through which tumor cells may specifically secrete circulating microRNAs. Such action may help modify the surroundings and create a favorable environment for tumor progression. In the current study, we observed that both miR-133a and mir-148b can be secreted by proliferating breast cancer cell lines. Although we do not know whether this observation is only limited to breast cancer cell lines or can also be observed in tumor tissues, our findings underscore the need to explore the underlying molecular mechanisms of circulating microRNAs.

Recently, The Cancer Genome Atlas (TCGA) data have been analyzed and released to the public. The breast cancer data in TCGA include a large number of primary breast tumors characterized by genomic DNA copy number arrays, DNA methylation, exome sequencing, mRNA arrays, and microRNA sequencing. Those data create a valuable resource for us to explore the biological functions of specific microRNAs. Although the available clinical information is still limited, we found that the expression of mir-148b was significant differed by histological types (P = 7.588×10^−12^ and q=3.29×10^−9^). In addition, in a recent analysis using TCGA RNA and small RNA data, Volinia et al found that mir-148b was among a panel of mRNAs/microRNAs which were associated with overall survival [57]. Although we did not observe similar associations for miR-133a, miR-133a has been reported to be a tumor suppressor and had prognostic value in esophageal squamous cell carcinoma [58, 59] and other cancers [60].

In addition to cancers, circulating microRNAs, especially inflammatory-related circulating microRNAs, may also be used as biomarkers for aging and other aging-related diseases [61, 62]. In a recent study by Noren Hooten et al, they found serum levels of miR-151a-5p, miR-181a-5p and miR-1248 were significantly decreased in 20 older individuals compared to 20 younger individuals [61]. In humans, miR-1248 was found to regulate the expression of mRNAs involved in inflammatory pathways and miR-181a was found to correlate negatively with the pro-inflammatory cytokines IL-6 and TNFα and to correlate positively with the anti-inflammatory cytokines TGFβ and IL-10. In addition, a number of circulating miRNAs, which are functionally related to proinflammatory, seem to be promising biomarkers for the major age-related diseases such as cardiovascular disease (CVD), type 2 diabetes mellitus (T2DM), Alzheimer Disease (AD), and rheumatoid arthritis (RA) [62].

In summary, we discovered and validated two circulating microRNAs, mir-148b and miR-133a, associated with early stage breast cancer. Since both microRNAs can be secreted from breast cancer cell lines, their existence in circulation may potentially serve as noninvasive screening and prognostic biomarkers for breast cancer. A large-scale prospective trial is needed to confirm their clinical usage.

## MATERIALS AND METHODS

### Study population

The study was approved by Institutional Research Board (IRB) of Roswell Park Cancer Institute (RPCI). Biosepcimens from RPCI were used as an initial discovery cohort. Anonymized biospecimens and questionnaire data used in this study were made available through the Data Bank and BioRepository (DBBR) of RPCI [63]. Patients are enrolled through site-specific clinics prior to surgery and chemotherapy, and controls are cancer-free individuals who are visitors or family members of patients, or are enrolled through community events. Relationships between patients and controls are carefully annotated, so to avoid overmatching patients to their own family or friends. Written consent is obtained prior to enrollment into the DBBR, which allows DBBR to provide anonymized biospecimens and questionnaire data for research (such as this study) without further consent. Patients and controls are consented to provide a non-fasting blood sample and to complete an epidemiological questionnaire. Blood samples are drawn in phlebotomy and transferred to the DBBR laboratory. Following DBBR standard operating procedure (SOP), samples are processed and blood components stored within one hour of collection to minimize degradation. Ten milliliters of whole blood was obtained from each study subject. EDTA-plasma was extracted by centrifuging whole blood at 3,000 rpm for 10 minutes at room temperature. All extracted plasma samples are stored in phased liquid nitrogen. To minimize the effect of freeze-thaw on circulating microRNAs, we only used plasma samples which had not been previously thawed. In this study, a total of 52 women with early stage invasive breast cancer (stage I and II), 35 women with DCIS, and 35 cancer-free women were included in the microRNA profiling analysis.

The validation cohort consisted of 50 early stage (stage I and II) breast cancer patients and 50 healthy controls who participated in a prospective case-control study for the molecular detection of breast cancer (MODE-B Study), conducted at the University Hospital Erlangen, Erlangen, Germany. Patients were newly diagnosed with breast cancer and pre-treatment blood samples were collected. For each patient, five tubes of peripheral blood (serum, plasma, PAXgene®, CPDA, EDTA), urine samples, fresh frozen tissue samples from the core needle biopsy at diagnosis and paraffin embedded tumor samples were available. An epidemiological questionnaire was completed via an in-person interview by cases and controls included in this study.

### Cell cultures

To determine the secretory potential of the significant microRNAs identified from microRNA profiling, two breast cancer cell lines, MCF-7 and MDA-231, were cultured and a fraction of the culture medium was collected at 0, 24 and 48 hours after the initial seeding of cells in 10-cm dish. 0.5 × 10^6^ cells were seeded in the initial dish.

### RNA isolation

Plasma and culture medium microRNAs were isolated using the miRNeasy kit (Qiagen) with minor modifications. In brief, 700 μl of QIAzol reagent was added to 400 μl of plasma sample or 1ml culture medium. The sample was mixed in a tube, followed by the addition of 3 μl of miSPIKE, spiked-in microRNA, at a concentration of 0.1 μM (IDT) and 140 μl of chloroform. After mixing vigorously for 15 s, the sample was then centrifuged at 12,000 g for 15 minutes. The upper aqueous phase was carefully transferred to a new collection tube, and 1.5 volume of ethanol was added. The sample was then applied directly to a silica membrane-containing column and the RNA was bound and cleaned using buffers provided by the manufacturer to remove impurities. The immobilized RNA was then collected from the membrane with a low salt elution buffer. Similar method was used to extract microRNAs from the cell culture medium. The quality and quantity of the RNA was evaluated by 260/280 ratio using NanoDrop spectrophotometry (NanoDrop ND-1000 Technologies Inc.) and Agilent 2100 Bioanalyzer (Agilent Technologies). The efficiency of small RNA isolation was monitored by the amount of spiked-in microRNA recovered by using PCR with sequence specific primers (IDT).

### microRNA profiling

In the discovery cohort, microRNA expression in the plasma samples was profiled using Exiqon MiRCURY LNA Universal RT microRNA PCR Technology, following the manufacturer's recommended protocol (Exiqon). The Serum/Plasma Focus microRNA PCR Panel is a microRNA-specific, LNA™-based system designed for sensitive and accurate detection of serum/plasma microRNA by quantitative real-time PCR using SYBR® Green. A total of 168 human microRNAs commonly found in human serum/plasma and 7 reference microRNAs were included in this panel. Only 20 ng total RNAs is needed in each analysis. Briefly, 20 ng total RNAs were reverse transcribed using the RT enzyme. The RT mixture was incubated for 60 min at 42˚C followed by heat-inactivation of the reverse transcriptase for 5 min at 95˚C. Multiplex RT reactions were diluted 62.5-fold with water, and 55μl of each diluted product was combined with 55μl of 2X Universal SYBR® Green master mix. One-hundred μl of the sample/master mix from each Multiplex pool was loaded into the array. Then, the array was centrifuged and mechanically sealed with the Applied Biosystems sealer device. qRT-PCR was carried out on an Applied BioSystems 7900HT real-time PCR instrument using the manufacturer's recommended cycling conditions. The experiment was performed at RPCI Genomic Core Shared Resource.

For the validation cohort, the expression levels of microRNAs were confirmed with a Taqman-based real-time quantitative PCR (RT-qPCR) using individual microRNA-specific primers and probes as described by the manufacturer (Applied Biosystems). The first-strand microRNA-cDNA PCR template was generated from 50 ng of total RNA according to the manufacturer's instructions. Approximately 2.5 ng of cDNA was then used in the PCR on a CFX96 Touch Real-Time PCR Detection System from Bio-Rad. Triplicate samples, validated endogenous controls, and inter-assay controls were used throughout. The qRT-PCR results were analyzed by SDS 2.2.2. To be consistent with the discovery cohort, we chose miR-93 as the endogenous control. RT-qPCR data were the normalized expression values in which the endogenous control miR-93 was used as the reference gene. For each assay, the Ct (Cycle threshold) of microRNA of interest in the TaqMan qPCR assay was subtracted from the average miR-93 Ct value to obtain a ΔCt value (miR-93 - microRNA of interest). A higher delta Ct value indicates a higher expression level of the microRNA of interest. For cell culture medium, similar qPCR was applied at baseline, 24 and 48 hours. For each assay at 24 and 48 hours, the Ct (Cycle threshold) of microRNA of interest in the TaqMan qPCR assay was subtracted from the baseline Ct value to obtain a ΔCt value.

### Data Analysis

For data quality control, we excluded samples with less than half of the profiled microRNAs with a Ct value less than 32. A total of 19 (out of 122) samples were removed, which left a total of 103 study subjects, including 47 patients with invasive breast cancer, 29 patients with DCIS, and 27 controls, for further analysis. We also excluded the list of blood cells derived microRNAs (including miR-16, miR-150, miR-486-5p, let-7a, miR-574-3p, miR-223, miR-197, miR-451, and miR-92a) from downstream analysis. The Exiqon's Serum/Plasma Focus microRNA PCR Panel supplies five microRNAs (miR-93, miR-103, miR-191, miR-423-3p, and miR-425) as candidate reference microRNAs for normalization. In our samples, miR-93 was the most stably expressed (Figure S1), so miR-93 was chosen as reference microRNA for normalization of the RT-PCT data.

Unsupervised hierarchical clustering algorithm based on the average linkage and Pearson correlation metric was performed based on the normalized expression profiles from the top 75% of the most variable (i.e., largest variance) microRNAs across 103 samples, as well as from all microRNAs [64]. We used the Limma program in the R-based Bioconductor package to calculate the statistical significance for the level of differential expression [65]. Briefly, a linear model was fit to the data, with cell means corresponding to the different conditions and a random effect for array. The Benjamini & Hochberg method was used to control the false discovery rate (FDR) for multiple testing [66].

## SUPPLEMENTARY FIGURE


