# Supplementary material for: Circulating miR-148b and miR-133a as biomarkers for breast cancer detection
Source: Oncotarget. 2014 May 26;5(14):5284–94. doi: 10.18632/oncotarget.2014 (PMC4170614; doi:10.18632/oncotarget.2014)
Supplement: Supplementary file 1 [file oncotarget-05-5284-s001.pdf]

Circulating miR-148b and miR-133a as biomarkers for breast cancer detection – Shen et al

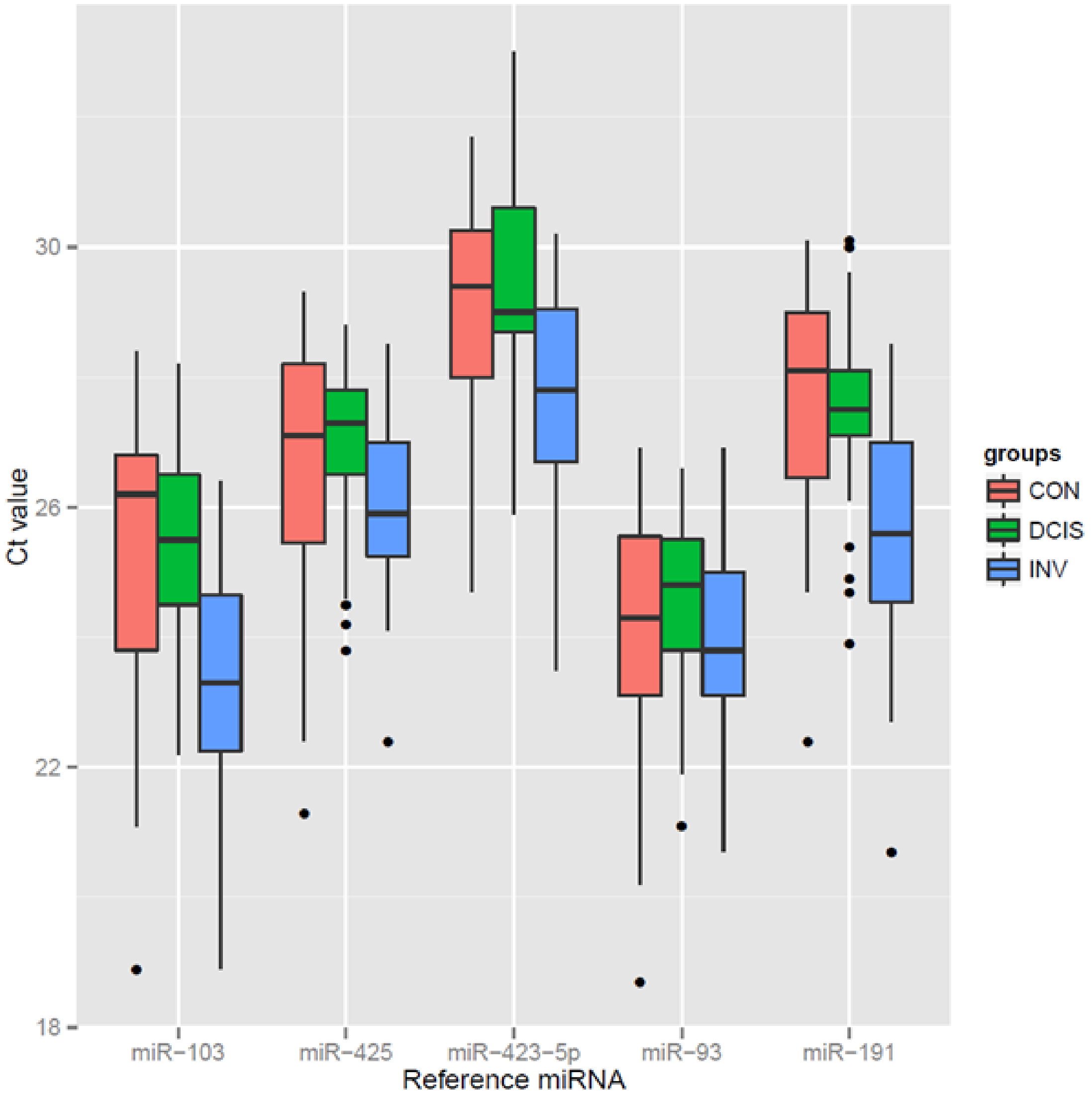

Figure S1: The distribution of Ct value for each of the five candidate reference microRNAs in control group (red), DCIS group (green) and invasive group (blue), respectively. The five candidate reference microRNAs are (from left to right): miR-103, miR-425, miR-423-5p, miR-93 and miR-191.
